# Determination of pressure properties of superconducting systems based on characteristic ratios 2Δ(0)/*T*_c_ and Δ*C*(*T*_c_)/*C*_N_(*T*_c_)

**DOI:** 10.1038/s41598-018-36733-1

**Published:** 2019-02-18

**Authors:** Mateusz Krzyzosiak, Ryszard Gonczarek, Adam Gonczarek, Lucjan Jacak

**Affiliations:** 1 University of Michigan-Shanghai Jiao Tong University Joint Institute, 800 Dongchuan Rd, Shanghai, 200240 China; 2 Faculty of Fundamental Problems of Technology, Wrocław University of Technology, Wybrzeże Wyspiańskiego 27, 50-370 Wrocław, Poland; 3 Faculty of Computer Science and Management, Wrocław University of Technology, Wybrzeże Wyspiańskiego 27, 50-370 Wrocław, Poland

## Abstract

A simplified analytical model of the effect of high pressure on the critical temperature and other thermodynamic properties of superconducting systems is developed using the general conformal transformation method and group-theoretical arguments. Relationships between the characteristic ratios ${ {\mathcal R} }_{1}\equiv 2{\rm{\Delta }}(0)/{T}_{{\rm{c}}}$ and ${ {\mathcal R} }_{2}\equiv {\rm{\Delta }}C({T}_{{\rm{c}}})/{C}_{{\rm{N}}}({T}_{{\rm{c}}})$ and the stability of the superconducting state is discussed. Including a single two-parameter fluctuation in the density of states, placed away from the Fermi level, stable solutions determined by ${ {\mathcal R} }_{1}$ are found. It is shown that the critical temperature *T*_c_(*p*), as a function of high external pressure, can be predicted from experimental data, based on the values of the two characteristic ratios, the critical temperature, and a pressure coefficient measured at zero pressure. The model can be applied to *s*-wave low-temperature and high-temperature superconductors, as well as to some novel superconducting systems of the new generation. The problem of emergence of superconductivity under high pressure is explained as well. The discussion is illustrated by using experimental data for superconducting elements available in the literature. A criterion for compatibility of experimental data is formulated, allowing one to identify incompatible measurement data for superconducting systems for which the maximum or the minimum critical temperature is achieved under high pressure.

## Introduction

Recent discoveries of new classes of superconducting materials, with the most prominent example of iron-based superconductors, and ongoing development of superconductivity-based devices in the fields such as quantum information processing and fast digital circuits have kept superconductivity in the spotlight on the condensed matter physics stage. The accessibility of high-pressure experimental techniques has resulted in an advancement of characterization methods, providing a useful insight into the nature of superconductivity at high pressures in a wide class of materials. The amount of experimental data available has been also pushing forward research efforts on the theoretical front. In particular, many properties of novel superconducting systems under high external hydrostatic pressure have been recently studied by *ab-initio* numerical calculations^1–11^. The results of these studies are usually in quite a good agreement of with the available experimental data. Being successful in providing quantitative characteristics of superconducting systems under high pressure, the *ab-initio* studies do not however provide much information about which of the system’s parameters and to what extent affect these characteristics and material properties.

In refs^12,13^ we developed a simple analytical model of the effect of high pressure on the critical temperature and other thermodynamic properties of superconductors. The model allowed us to identify four general types of superconductors, based on the features of the *T*_c_ vs. pressure characteristics. The distinct behaviour of these four classes of superconductors can be studied with respect to the form of the density of states, which is determined within the so-called general conformal transformation method, taking into account fundamental properties and symmetry of the superconducting system.

In the present paper we illustrate the versatility of that model, by determining some properties of superconducting system based on experimental data. Within the model it is possible to study the characteristic ratio ${ {\mathcal R} }_{1}\equiv 2{\rm{\Delta }}\mathrm{(0)/}{T}_{{\rm{c}}}$, that is a relation between the energy gap Δ at *T* = 0 and the transition temperature *T*_c_, and another characteristic ratio ${ {\mathcal R} }_{2}\equiv {\rm{\Delta }}C({T}_{{\rm{c}}})/{C}_{{\rm{N}}}({T}_{{\rm{c}}})$, where Δ*C*(*T*_c_) = *C*_S_(*T*_c_) − *C*_N_(*T*_c_), defines the jump of the heat capacity between the superconducting and the normal phase at the transition temperature. The fundamental thermodynamic quantities defining the ratios ${ {\mathcal R} }_{1}$ and ${ {\mathcal R} }_{2}$, and hence the ratios themselves, can be measured using experimental techniques and are widely reported in the literature^5,14–23^. Therefore experimental results can be easily compared against theoretical predictions, in a way that is presented in this paper.

Because of intrinsic uncertainty of experimental methods, the experimentally found values of material parameters have limited accuracy, usually of the order of a few percent. Moreover, different experimental techniques may provide different estimates for the same parameter. However, these fluctuations are again usually of the order of a few percent. Therefore, the numerical results given below, although derived within the specified precision, should be treated as estimates and may be subject to minor changes, depending on the precision of the experimental data available.

## Method

Numerous properties of various classes of superconductors, including *s*-wave low-temperature and high-temperature superconductors, as well as superconducting systems on the new generation, can be studied by within a simple analytical model, based on the general conformal transformation method and justified using group-theoretical arguments^13,24–32^.

### Conformal transformation method

The general conformal transformation method allows us to transfer any model of a superconducting system formulated in the original reciprocal (momentum) space to a mathematically fully equivalent model in an isotropic reciprocal space. The fact that the space becomes isotropic, does not mean that the method simply neglects some degrees of freedom: They are instead encoded in an involved function of two (or three) variables, that in the conformal transformation method replaces the usual electronic density of states. All properties of the system included originally in the dispersion relation, are transferred to this new function – a scalar field of the density of states.

More formally, applying the conformal transformation with elements of the group theory, we arrive at a mathematically equivalent description in an isotropic reciprocal space. The symmetry of the superconducting energy gap follows then from a requirement of choosing an appropriate subspace of irreducible representations, yielding a scalar field of the density of states. This field, for a trivial representation — corresponding to the *s*-wave symmetry — reduces to the standard electronic density of states. In other, more complicated scenarios, for *T* = *T*_c_ (and Δ = 0), the scalar density of states is averaged with the spherical harmonics (for 3D systems) or the Fourier harmonics (in the 2D case). This procedure, eventually, also yields a density-of-states-type function. Consequently, the pairing potential, which in general has a spin-antisymmetric or a spin-symmetric structure, becomes expressed in terms of a double series of the spherical harmonics (or the Fourier harmonics) indexed by the number *l* for 3D (or 2D) systems. For the spin-antisymmetric part, only the harmonics with even values of *l* are included, whereas for the spin-symmetric part — only those with odd values. Finally, it is enough to take *l* = 0 and assume the pairing interaction amplitude *g* to be just a constant function. Then the scalar field of the density of states reduces to the density of states.

The density of states and the averaged density-of-states-type function are quite complicated functions, but in general we expect them to feature some fluctuations. The fluctuations can be either positive (peaks) or negative (valleys). The case that we have discussed in our previous paper^33^ and also discuss in the present work, is that of a density of states featuring a single two-parameter Dirac-*δ* type fluctuation, placed away from the Fermi level. This fluctuation (a peak) is imposed onto an otherwise constant-value density of states characteristic to the BCS model. This simplification makes an arbitrary model of a superconductor to eventually correspond to a model of a *s*-wave superconductor. As we argue above, it is a natural consequence of the used mathematical formalism and not an *a priori* assumption (which would have been incorrect).

### Pressure effects

The method we use to include the hydrostatic pressure in our model is presented and discussed in detail in ref.^33^. Therefore, in this subsection we just recall its main idea: The approach starts out from a set of two equations for a pressure-free system (*p* = 0). One of these equations is the superconducting gap equation, derived within the Green function formalism in the mean-field approximation. Another self-consistent equation for the carrier concentration completes the set and brings about equations describing the thermodynamic properties of the superconductor taking into account external pressure.

External pressure imposed on a superconducting sample leads to a stable configuration under new conditions. In particular, the new equilibrium yields a modified dispersion relation for charge carriers, parametrized now by the external pressure. More specifically, high pressure, applied to a superconducting sample, supplies an extra amount of energy to each unit cell. The amount of that additional energy is is absorbed by unit cell atoms, and hence a new equilibrium state is reached with the dispersion relation, parametrized also by the pressure *p*.

In the following sections we assess the impact of high pressure on the critical temperature, based on experimental data for the characteristic ratios ${ {\mathcal R} }_{1}$ and ${ {\mathcal R} }_{2}$ for various superconductors, and using results obtained within the above outlined conformal transformation method^13,24–33^. In particular, we discuss the relationship between the values of the characteristic ratios ${ {\mathcal R} }_{1}$, ${ {\mathcal R} }_{2}$ and the stability of the superconducting state. We find stable solutions for superconducting systems determined by these characteristic ratios and some additional restrictions.

In order to describe pressure effects within the conformal transformation method, we assume that the peak in the density of states may be located either above or below the Fermi level, at the point *X*_0_ = 2*T*_c_*x*_0_. The dimensionless density of states itself is taken in the form ${\mathscr{N}}(\xi )=1+2\chi \delta (x-{x}_{0})$, where the parameter *χ* is the height of the local fluctuation, that can be either positive (corresponding to a peak) as well as negative (a valley). As we show in the paper, within our model we are able to reproduce experimentally observed *T*_c_ vs pressure dependence for various superconducting materials. This suggests that it is the fluctuations (peaks/valleys) in the DOS, emerging in the conformal transformation method, that imply the type of the *T*_c_ vs pressure response.

## Critical Temperature and Characteristic Ratios for a Pressure-Free System

### Critical temperature

Within the model featuring a single fluctuation in the density of states function of form 2*χδ*(*x* − *x*_0_), the critical temperature *T* = *T*_c_(*χ*, *x*_0_), when Δ = 0, can be found as^33^

1 $${T}_{{\rm{c}}}(\chi ,{x}_{0})={T}_{{\rm{c}}}(0,0)\,\exp (\chi \,\frac{\tanh \,{x}_{0}}{{x}_{0}})\,,$$

where *T*_c_(0, 0) is the critical temperature in the standard BCS-type model, with

2 $${T}_{{\rm{c}}}\mathrm{(0},\mathrm{0)}=\frac{{\xi }_{{\rm{p}}}}{{k}_{{\rm{B}}}}\,\exp (-\,\frac{1}{{\nu }_{0}g}).$$

The so-called cut-off parameter *ξ*_p_ in formula (2) is determined individually for each superconducting system and depends on details of the pairing mechanism^13^. For systems with an electron-phonon pairing potential the cut-off parameter corresponds to the Debye energy *k*_B_*T*_D_, where *k*_B_ is the Boltzmann’s constant and *T*_D_ denotes the Debye temperature. The dimensionless amplitude of the pairing interaction is *ν*_0_*g*, with *ν*_0_ being the density of states of the BCS type.

### Ratios ${ {\mathcal R} }_{1}$ and ${ {\mathcal R} }_{2}$

The characteristic ratio ${ {\mathcal R} }_{1}(\chi ,{x}_{0})=2{\rm{\Delta }}(0,\chi ,{x}_{0})/{T}_{{\rm{c}}}(\chi ,{x}_{0})$ as a function *χ* and *x*_0_ is found from the following equation^33^

3 $${ {\mathcal R} }_{1}(\chi ,{x}_{0})={ {\mathcal R} }_{1}^{\ast }\exp \{\chi [\frac{4}{\sqrt{{\mathrm{(4}{x}_{0})}^{2}+{ {\mathcal R} }_{1}^{2}(\chi ,{x}_{0})}}-\,\frac{\tanh \,{x}_{0}}{{x}_{0}}]\},$$

where ${ {\mathcal R} }_{1}^{\ast }=2\pi {e}^{-C}$ is the characteristic ratio ${ {\mathcal R} }_{1}$ for the BCS case (*χ* = 0), and *C* denotes the Euler constant. The ratio ${ {\mathcal R} }_{1}(\chi ,{x}_{0})$ is found numerically, assuming that $2\le { {\mathcal R} }_{1}(\chi ,{x}_{0})\le 6$.

The other characteristic ratio ${ {\mathcal R} }_{2}\equiv {\rm{\Delta }}C({T}_{{\rm{c}}})/{C}_{{\rm{N}}}({T}_{{\rm{c}}})$, where Δ*C*(*T*_c_) = *C*_S_(*T*_c_) − *C*_N_(*T*_c_), quantifying the jump of the heat capacity between the superconducting and the normal phase at the transition temperature, is also a function of *χ* and *x*_0_. Referring again to results presented in ref.^33^, the value of the ratio can be found as

4 $${ {\mathcal R} }_{2}(\chi ,{x}_{0})=\frac{6\,\exp (\chi \,\frac{\tanh \,{x}_{0}}{{x}_{0}})}{{\pi }^{2}a[1+\frac{\chi }{3a}\,\phi ({x}_{0})]},$$

where *a* = 7*ζ*(3)/2*π*^2^ and $\phi (x)=\frac{3}{2{x}^{3}}(\tanh \,x-x\,{\cosh }^{-2}x)$, with *ζ*(*n*) denoting the the Riemann-*ζ* function. Note that *ζ*(3) = 1.202056 …, and $\phi (x)\le 1$ is an even and positive function of the real variable *x*. Moreover, *φ*(0) = 1 and it quickly approaches 0 as |*x*| increases from 0 to ∞.

From Eq. (3), the parameter *χ* can be found in terms of ${ {\mathcal R} }_{1}$ and *x*_0_, and substituted into Eq. (4). Eventually, the ratio ${ {\mathcal R} }_{2}$ as a function of ${ {\mathcal R} }_{1}$ and *x*_0_ is found as

5 $$\begin{array}{rcl}{ {\mathcal R} }_{2} & = & \frac{6}{{\pi }^{2}\,a}\exp [\frac{\tanh \,{x}_{0}}{{x}_{0}}\,\frac{\sqrt{{\mathrm{(4}{x}_{0})}^{2}+{ {\mathcal R} }_{1}^{2}}\,\mathrm{ln}({ {\mathcal R} }_{1}/{ {\mathcal R} }_{1}^{\ast })}{4-\frac{\tanh \,{x}_{0}}{{x}_{0}}\,\sqrt{{\mathrm{(4}{x}_{0})}^{2}+{ {\mathcal R} }_{1}^{2}}}]\\  &  & \times {[1+\frac{\phi ({x}_{0})\sqrt{{\mathrm{(4}{x}_{0})}^{2}+{ {\mathcal R} }_{1}^{2}}\mathrm{ln}({ {\mathcal R} }_{1}/{ {\mathcal R} }_{1}^{\ast })}{3a[4-\frac{\tanh {x}_{0}}{{x}_{0}}\sqrt{{(4{x}_{0})}^{2}+{ {\mathcal R} }_{1}^{2}}]}]}^{-1},\end{array}$$

where ${ {\mathcal R} }_{1}^{\ast }=3.527756\ldots ,$ *a* = 0.426278 …, $\frac{6}{{\pi }^{2}a}=1.426104\ldots$. The formula (5), in the specific case *x*_0_ = 0, has proved to provide a good fit to experimental data for some low-temperature superconducting materials^24^. Note that the right-hand sides of expressions (1), (3), (4), and (5) are all even functions of *x*_0_. This allows us to use Eq. (5) to derive just |*x*_0_|, when ${ {\mathcal R} }_{1}$ and ${ {\mathcal R} }_{2}$ are known from experimental data.

We supplement the set of equations for a pressure-free system with a formula for the free energy difference Δ*F*. The formula, derived for the sub-critical temperature range, *i.e*. for $T\lesssim {T}_{{\rm{c}}}(\chi )$, in the first order of the perturbation method, has the form^33^

6 $${\rm{\Delta }}F(T,\chi ,{x}_{0})=-\,\frac{{\nu }_{0}{T}_{{\rm{c}}}^{2}(\chi ,{x}_{0})}{a[1+\frac{\chi }{3a}\,\phi ({x}_{0})]}\,{[1-\frac{T}{{T}_{{\rm{c}}}(\chi ,{x}_{0})}]}^{2}.$$

Note that a system can be in the superconducting state only if ${\rm{\Delta }}F(T,\chi ,{x}_{0}) < 0$, and it occurs if $1+\frac{\chi }{3a}\,\phi ({x}_{0}) > 0$.

### Forms of the characteristic ratio ${ {\mathcal R} }_{1}(\chi ,{x}_{0})$

**Figure 1:**
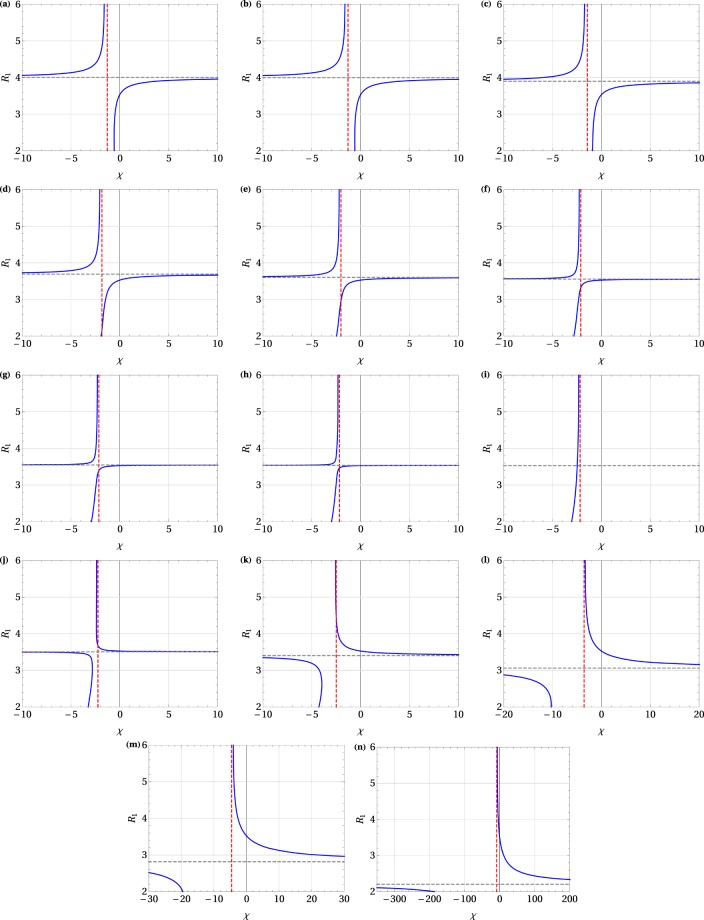
Ratio ${ {\mathcal R} }_{1}$ as a function of the parameter *χ* for selected values of *x*_0_: (**a**) 0, (**b**) ±0.1, (**c**) ±0.4, (**d**) ±0.7, (**e**) ±0.8, (**f**) ±0.85, (**g**) ±0.86, (**h**) ±0.87, (**i**) ±0.8791, (**j**) ±0.9, (**k**) ±1, (**l**) ±1.3, (**m**) ±1,5, (**n**) ±2.

In this subsection we discuss solutions to Eq. (3) for several selected values of *x*_0_. We find ${ {\mathcal R} }_{1}(\chi ,{x}_{0})$, which is an even function of *x*_0_, numerically in dependence on *χ*. The solutions, shown in Fig. 1 and discussed in detail later in this section, have a common feature: there are two separate curves in each graph, with a horizontal asymptote ${ {\mathcal R} }_{1}={ {\mathcal R} }_{A}({x}_{0})$, where

7 $${ {\mathcal R} }_{A}(x)=\frac{4\,x}{\sinh \,x},$$

and $${ {\mathcal R} }_{A}({x}_{0})={ {\mathcal R} }_{1}^{\ast }$$ for $${x}_{0}\equiv {x}_{0}^{\ast }=\pm 0.879077\ldots $$.

Superconductivity in the sub-critical temperature range ($T\lesssim {T}_{{\rm{c}}}(\chi ,{x}_{0})$) is realized only if the free energy difference between the superconducting and the normal state is negative. Otherwise, the system is in the normal phase. According to formula (6), the free energy difference is positive if $\chi  < {\chi }_{s}({x}_{0})$, with

8 $${\chi }_{s}(x)=-\,\frac{3\,a}{\phi (x)},$$

and hence this inequality defines unstable regions, where superconductivity is suppressed. Examining Eq. (3) for |*x*_0_| ≤ 0.288, we find that for stable solutions (the values of ${ {\mathcal R} }_{1}(\chi ,{x}_{0})$) to exist, the condition $\chi \ge {\chi }_{m}({x}_{0})$ must be satisfied. For *χ* = *χ*_*m*_(*x*_0_), the values of ${ {\mathcal R} }_{t}({x}_{0})\equiv { {\mathcal R} }_{1}({\chi }_{m}({x}_{0}),{x}_{0})$ must be derived from the equation

$${ {\mathcal R} }_{t}={ {\mathcal R} }_{1}^{\ast }\exp \{\frac{16{x}^{2}[\tanh \,x\,{(1+\frac{{ {\mathcal R} }_{t}^{2}}{16{x}^{2}})}^{\mathrm{3/2}}-\,1]}{{ {\mathcal R} }_{t}^{2}}-\,1\},$$

and, thereafter, *χ*_*m*_(*x*_0_) is found as

9 $${\chi }_{m}(x)=\,\mathrm{ln}\,\frac{{ {\mathcal R} }_{t}(x)}{{ {\mathcal R} }_{1}^{\ast }}\,{[\frac{4}{\sqrt{16{x}^{2}+{ {\mathcal R} }_{t}^{2}(x)}}-\frac{\tanh x}{x}]}^{-1}.$$

**Figure 2:**
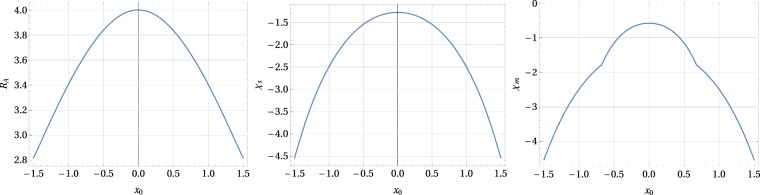
The values of ${ {\mathcal R} }_{A}$ (defining horizontal asymptotes), the vertical cut-off line *χ*_*s*_ (delineating unstable regions), and the minimum value of *χ* ≡ *χ*_*m*_ for which superconducting state can be realized, are shown here as functions of *x*_0_.

Note that both expressions: the one for ${ {\mathcal R} }_{t}(x)$, as well as that for *χ*_*m*_(*x*) are even functions of *x*. This estimate can be extended by the minimum value *χ* that is achieved for ${ {\mathcal R} }_{1}=2$, when −0.677 ≤ *x*_0_ ≤ −0.288 and 0.288 ≤ *x*_0_ ≤ 0.677. Then *χ*_*m*_(*x*_0_) is given by Eq. (9) with ${ {\mathcal R} }_{t}({x}_{0})\equiv 2$. In particular, we have *χ*_*m*_(0) = −0.579, ${ {\mathcal R} }_{t}\mathrm{(0)}=2.315$, *χ*_*m*_(0.1) = −0.597, ${ {\mathcal R} }_{t}\mathrm{(0.1)}=2.281$, *χ*_*m*_(0.2) = −0.654, ${ {\mathcal R} }_{t}\mathrm{(0.2)}=2.157$, *χ*_*m*_(0.4) = −0.928, ${ {\mathcal R} }_{t}\mathrm{(0.4)}=2$, and *χ*_*m*_(0.6) = −1.473, ${ {\mathcal R} }_{t}\mathrm{(0.6)}=2$. For $|{x}_{0}| > 0.677$ the minimum allowed value of *χ* is determined by *χ*_*s*_(*x*_0_), and then *χ*_*m*_(*x*_0_) ≡ *χ*_*s*_(*x*_0_). The graph of *χ*_*m*_ as a function of *x*_0_ is shown in Fig. 2.

The graphs of the ratio ${ {\mathcal R} }_{1}$ vs *χ* for selected values of *x*_0_ are shown in Fig. 1. Black dashed lines in the graphs indicate the horizontal asymptotes ${ {\mathcal R} }_{1}={ {\mathcal R} }_{A}({x}_{0})$, and unstable regions are cut off by the vertical red dashed lines *χ* = *χ*_*s*_. Only the red line in the graphs defines stable solutions. However, for the graphs in Fig. 1(d–h) the solutions ${ {\mathcal R} }_{1} > { {\mathcal R} }_{t}$, and for the graphs in Fig. 1(j,k) the solutions ${ {\mathcal R} }_{1} > { {\mathcal R} }_{t}$ are unstable, and therefore superconductivity is suppressed. Hence, ${ {\mathcal R} }_{t}$ defines a stability threshold for these solutions. Details of the positions of all characteristic lines for graphs presented in Fig. 1 are given in Table 1.

**Table 1:** Positions of horizontal asymptotes ${ {\mathcal R} }_{1}={ {\mathcal R} }_{A}$ and vertical cut-off lines *χ* = *χ*_*s*_ for unstable regions in Fig. 1(a–n). Stability threshold values ${ {\mathcal R} }_{t}$ are also given.

*x*_0_	${{\boldsymbol{ {\mathcal R} }}}_{{\boldsymbol{A}}}$	*χ*_*s*_	${{\boldsymbol{ {\mathcal R} }}}_{{\boldsymbol{t}}}$	Figure 1	*x*_0_	${{\boldsymbol{ {\mathcal R} }}}_{{\boldsymbol{A}}}$	*χ*_*s*_	${{\boldsymbol{ {\mathcal R} }}}_{{\boldsymbol{t}}}$	Figure 1
0	4	−1.279	—	(a)	±0.87	3.537	−2.165	3.462	(h)
±0.1	3.993	−1.289	—	(b)	±0.8791	3.5278	−2.1856	3.5278	(i)
±0.4	3.985	−1.447	—	(c)	±0.9	3.507	−2.235	3.679	(j)
±0.7	3.691	−1.827	2.173	(d)	±1	3.404	−2.496	4.455	(k)
±0.8	3.603	−2.013	2.943	(e)	±1.3	3.062	−3.554	—	(l)
±0.85	3.556	−2.120	3.316	(f)	±1.5	2.818	−4.538	—	(m)
±0.86	3.546	−2.142	3.389	(g)	±2	2.206	−8.290	—	(n)

Moreover, Fig. 1(i) refers to the case ${ {\mathcal R} }_{A}({x}_{0})={ {\mathcal R} }_{1}^{\ast }$, for which *x*_0_ has been derived by means of Eq. (7). For this case, no stable solution can exist at all. In our discussion, based on experimental data, we assume that $2\le { {\mathcal R} }_{1}\le 6$. However, the analysis can be extended beyond these limits.

In Table 2 the values of model parameters |*x*_0_|, *χ*, ${ {\mathcal R} }_{A}$, and *χ*_*s*_ for some superconducting materials are given. They have been found based on the experimental data (*T*_c_, ${ {\mathcal R} }_{1}$, and ${ {\mathcal R} }_{2}$) for a number of low-temperature superconducting materials. We also include the values of *T*_0_ (the critical temperature) and *ν*_0_*g* (the dimensionless pairing parameter) determined within the standard BCS-model.

**Table 2:** Experimental data *T*_c_ = *T*_c_(*χ*, *x*_0_, 0), ${ {\mathcal R} }_{1}(\chi ,{x}_{0})$, ${ {\mathcal R} }_{2}(\chi ,{x}_{0})$, ${T}_{{\rm{c}}}^{{\rm{\max }}}={T}_{{\rm{c}}}(\chi ,{x}_{0},{p}_{m})$ and *p*_*m*_ of some superconducting (SC) materials. The corresponding values of |*x*_0_|, *χ*, ${ {\mathcal R} }_{A}$ and *χ*_*s*_, derived within the model are given, and the possible type of the *T*_c_ vs pressure dependence is indicated. Additionally, the values of *ν*_0_*g*, and *T*_0_ = *T*_c_(0, 0) derived for a given *T*_D_ (the Debye temperature) are included^5,19,21,39,40^. The data for Nb ^(1)^ is taken from refs^15,22^, and that for Nb^(2)^ from ref.^43^.

SC	*T*_c_	${{\boldsymbol{ {\mathcal R} }}}_{{\bf{1}}}$	${{\boldsymbol{ {\mathcal R} }}}_{{\bf{2}}}$	${{\boldsymbol{T}}}_{{\bf{c}}}^{{\bf{\max }}}$	*p*_*m*_	|*x*_0_|	*χ*	${{\boldsymbol{ {\mathcal R} }}}_{{\boldsymbol{A}}}$	*χ*_*s*_	Type	*T*_D_	*T*_0_	*ν*0*g*
material	[K]	[1]	[1]	[K]	[GPa]	[1]	[1]	[1]	[1]		[K]	[K]	[1]
Al	1.20	3.3	1.4	—	—	0,710	−1.138	3.683	−1.844	—	428	3.19	0.204
Cd	0.52	3.2	1.4	—	—	0.605	−0.943	3.766	−1.679	—	209	1.21	0.194
Hg (*α*)	4.15	4.6	2.4	—	—	1.175	−2.804	3.209	−3.058	—	71.9	29.8	1.135
In	3.4	3.6	1.7	—	—	0.528	0.483	3.820	−1.579	—	108	2.18	0.256
Nb ^(0)^	9.26	3.8	1.9	9.7	4.5	0.982	−2.073	3.423	−2.446	(D)	275	45.5	0.556
Nb^(1)^	9.26	3.6	1.87	9.95	10	0.600	0.689	3.770	−1.672	(A)	275	5.00	0.250
Nb^(2)^	9.13	3.6	1.87	9.82	30	0.600	0.689	3.770	−1.672	(A)	275	4.93	0.249
Pb	7.19	4.3	2.7	—	—	1.092	−2.543	3.303	−2.773	—	105	46.1	1.215
Sn	3.72	3.5	1.6	5.3	11.3	0.866	−1.645	3.541	−2.155	(D)	200	14.0	0.377
Ta	4.41	3.6	1.6	4.5	40	0.436	0.345	3.876	−1.480	(A)	240	3.18	0.231
Tl	2.39	3.6	1.5	—	—	0.080	0.186	3.996	−1.285	—	78.5	1.98	0.272
V	5.38	3.4	1.5	16.5	120	0.807	−1.445	3.597	−2.028	(D)	380	16.6	0.320
Zn	0.855	3.2	1.6	—	—	0.714	−1.320	3.679	−1.851	—	237	2.66	0.223

Note that the values of *χ* for Hg (*α*) and Pb are quite close to the boundary of unstable regions delineated by *χ*_*s*_. Therefore, if *x*_0_ < 0, then as we will discuss in the following Section 4, applying external pressure to the superconducting system turns it unstable and superconductivity is suppressed.

## Effects of External Hydrostatic Pressure

### Response to external pressure: universal types of *T*_c_ vs pressure dependence

As we have shown in ref.^24^ the inclusion of external pressure *p* in Eqs (1), (3–5) results in the variable *x*_0_ being replaced by ${x}_{0}\to {\tilde{x}}_{0}=\tau ({x}_{0}+\kappa p),$ where

$$\tau \equiv \tau (\chi ,{x}_{0},p)=\frac{{T}_{{\rm{c}}}(\chi ,{x}_{0},\,\mathrm{0)}}{{T}_{{\rm{c}}}(\chi ,{x}_{0},p)},$$

and $\kappa  > 0$ is the coefficient of linear expansion in *p*. Then, from Eq. (1), we can find

10 $${T}_{{\rm{c}}}(\chi ,{x}_{0},p)={T}_{{\rm{c}}}(\chi ,{x}_{0},\,0)\,\exp \{\chi \,[\frac{\tanh \,[\tau ({x}_{0}+\kappa p)]}{\tau ({x}_{0}+\kappa p)}-\,\frac{\tanh \,{x}_{0}}{{x}_{0}}]\}.$$

In order to illustrate the discuss and predict the critical temperatures vs. pressure characteristics for superconducting systems under pressure, we will use Eq. (10), which after some algebra takes the form

$$t=\exp \{\chi [\frac{t\,\tanh \,[{t}^{-1}(\alpha \mp \mathrm{1)}{x}_{0}]}{(\alpha \mp \mathrm{1)}{x}_{0}}-\,\frac{\tanh \,{x}_{0}}{{x}_{0}}]\},$$

where

$$t=\frac{{T}_{{\rm{c}}}(\chi ,{x}_{0},p)}{{T}_{{\rm{c}}}(\chi ,{x}_{0}\mathrm{,0)}}\equiv \frac{{T}_{{\rm{c}}}(p)}{{T}_{{\rm{c}}}\mathrm{(0)}}$$

and *α* = *κp*/|*x*_0_| is a dimensionless positive scaling parameter for pressure. The upper sign in “∓” must be taken for the cases with ${x}_{0} < 0$, whereas the lower one for ${x}_{0} > 0$. Nte that if ${x}_{0} < 0$, there is a characteristic value of pressure *p*_*m*_ = |*x*_0_|/*κ*, corresponding to *α* = 1, for which the maximum or the minimum critical temperature is achieved.

**Figure 3:**
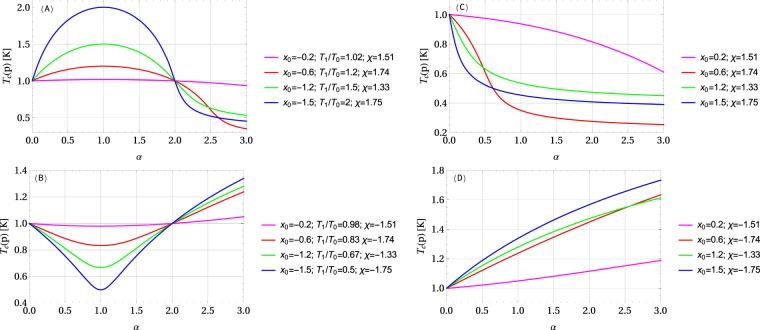
Four possible types of the dependence of the critical temperature on high external pressure. Here *α* = *κp*/|*x*_0_| is a dimensionless positive scaling parameter for pressure: (**A**) ${T}_{{\rm{c}}}\mathrm{(1)} > {T}_{{\rm{c}}}\mathrm{(0)}$ and ${x}_{0} < 0$, then $\chi  > 0$, (**B**) ${T}_{{\rm{c}}}\mathrm{(1)} < {T}_{{\rm{c}}}\mathrm{(0)}$ and ${x}_{0} < 0$, then $\chi  < 0$, (**C**) only *T*_c_(0) is given, ${x}_{0} > 0$ and $\chi  > 0$, (**D**) only *T*_c_(0) is given, ${x}_{0} > 0$ and $\chi  < 0$. It is shown that the shape of the curves changes, depending on the parameters *x*_0_ and *χ*.

The simple analytical model of the effect of high pressure on the critical temperature and other thermodynamic properties of superconductors presented in ref.^33^, allowed us to identify four possible types of the dependence of the critical temperature *T*_c_ on high external pressure *p*. Namely, for ${x}_{0} < 0$ the critical temperature *T*_c_(*χ*, *x*_0_, *p*) achieves a maximum for some $\chi  > 0$, and a minimum for $\chi  < 0$, if *p* = −*x*_0_/*κ*. On the other hand, if *x*_0_ ≥ 0 the applied pressure drives the critical temperature downwards *T*_c_(*χ*, *x*_0_, *p*) ≤ *T*_c_(*χ*, *x*_0_, 0) if $\chi  > 0$, or upwards *T*_c_(*χ*, *x*_0_, *p*) ≥ *T*_c_(*χ*, *x*_0_, 0) if $\chi  < 0$. Consequently, for the four possible combinations of parameters ${x}_{0} < 0$, ${x}_{0} > 0$ and $\chi  > 0$, $\chi  < 0$, four different types of the response to the applied pressure are possible, as far as the critical temperature is concerned. These four cases are illustrated in Fig. 3. However, let us emphasize that Fig. 3 illustrates just several examples of possible examples of the response for some given values *x*_0_ and *χ*, as the shape of the curves changes with different values of the critical temperature or with pressure scaling. In particular for the type-(A) and the type-(B) response, the dimensionless positive scaling parameter *α* = *κp*/|*x*_0_|, and *α* = 1 corresponds to the pressure giving the maximum or the minimum critical temperature, respectively.

For many superconducting systems, it is enough to take 0 ≤ *α* ≤ 3 and scale it with the appropriate values of pressure^33^. On the other hand, for the type-(C) and the type-(D) behavior, the dimensionless positive scaling parameter *α* can be scaled at will if ${x}_{0} > 0$, and it cannot be defined if *x*_0_ = 0. Hence, for some cases ${x}_{0} > 0$, it may occur that *α* = *κp*/*x*_0_ runs into values of the order of 100. Examples of such materials are: NaCoO a type-(C) superconductor^34^, with *T*_c_ = 4.68 K at *p* = 0, *T*_c_ = 4.25 K at *p* = 1.5 GPa modelled with *x*_0_ = 0.01 and *χ* = 1 for which ${ {\mathcal R} }_{1}=3.760$ and ${ {\mathcal R} }_{2}=0.939$, TlBaCuO being a type-(C) superconductor^35^, with *T*_c_ = 40 K at *p* = 0, *T*_c_ = 25 K at *p* = 1.76 GPa, modelled with *x*_0_ = 1 and *χ* = 1.6 for which ${ {\mathcal R} }_{1}=3.485$ and ${ {\mathcal R} }_{2}=1.343$. NdFeAsOF a type-(C) superconductor^36^, with *T*_c_ = 45.4 K at *p* = 0, *T*_c_ = 25 K at *p* = 9.4 GPa modelled with *x*_0_ = 0.84 and *χ* = 1.68 for which ${ {\mathcal R} }_{1}=3.544$ and ${ {\mathcal R} }_{2}=1.677$, and the vanadium which is of the type (D). Another example of a composed superconductor is YBaCuO — a type-(A) superconductor where a few different behaviors can be observed^37^. Here we focus on two cases: *T*_c_ = 17.1 K at *p* = 0, *T*_c_ = 23.8 K at *p* = 4.1 GPa and *c*_*p*_ = 2.1 K/GPa with *x*_0_ = −1.585, *χ* = 0.787, for which ${ {\mathcal R} }_{1}=3.456$ and ${ {\mathcal R} }_{2}=1.971$, and *T*_c_ = 17.1 K at *p* = 0, *T*_c_ = 45 K at *p* = 18 GPa and *c*_*p*_ = 20 K/GPa with *x*_0_ = −1.629, *χ* = 2.242, for which ${ {\mathcal R} }_{1}=3.356$ and ${ {\mathcal R} }_{2}=3.009$, where based on a series of experimental data, we used the formulas given in ref.^33^, Eqs (3) and (5).

Some other superconductors reveal their high pressure properties in the region of small *α* (of the order of 0.1 or 1). Examples of such systems include: niobium, zinc, tin, and SmBaCuO^38^, where for the latter one *T*_c_ = 78.5 K at *p* = 0, *T*_c_ = 86.3 K at *p* = 2 GPa, *x*_0_ = 1 and *χ* = −1.05 for which ${ {\mathcal R} }_{1}=3.595$ and ${ {\mathcal R} }_{2}=0.978$. All of them are type-(D) materials. Another example of a low-*α* system is the type-(C) cadmium.

**Figure 4:**
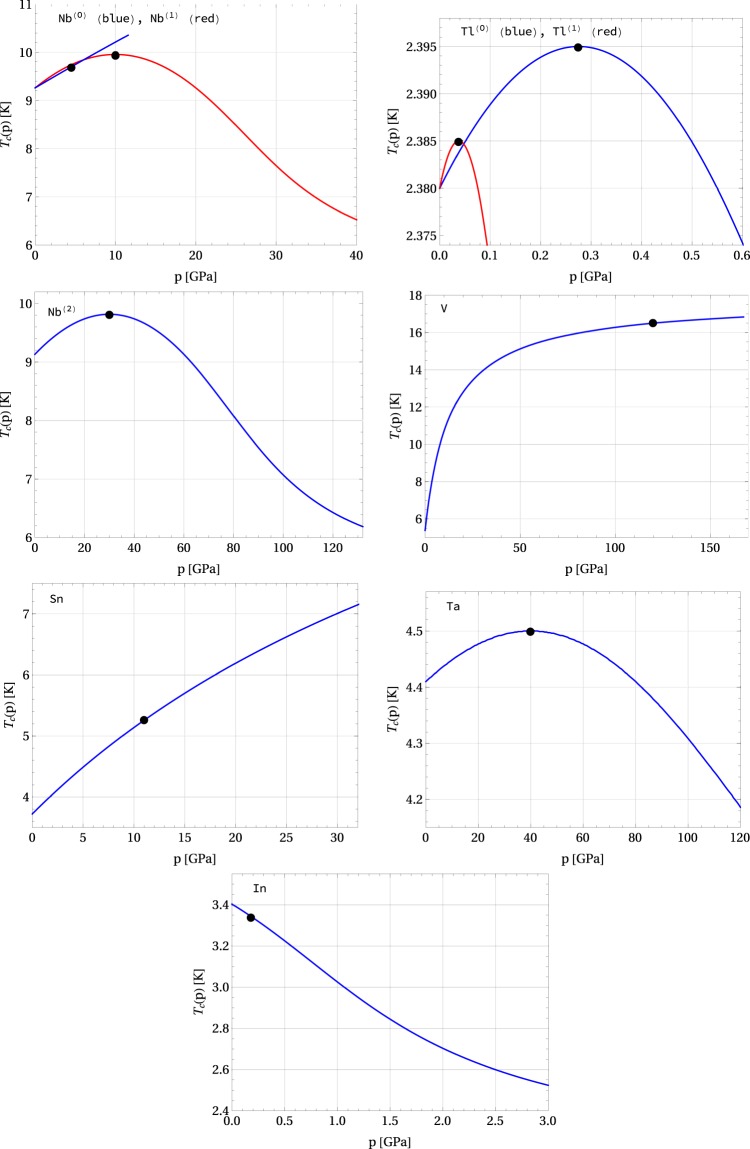
Critical temperature vs. pressure for selected superconductors: Nb^(0)^, Nb^(1)^, Nb^(2)^, Tl^(0)^, Tl^(1)^, V, Sn, Ta, and In. Black filled circles (●) indicate the experimental values listed in Table 2 for Nb^(0)^ (the critical temperature of 9.7 K at the pressure 4.5 GPa), Nb^(1)^ (9.95 K at 10 GPa), and Nb^(2)^ (9.82 K at 30 GPa), V (16.5 K at 120 GPa), Sn (5.3 K at 11.3 GPa), and Ta (4.5 K at 40 GPa). The data for Tl^(0)^ (2.385 K at 0.0377 GPa) and Tl^(1)^ (2.395 K at 0.275 GPa) is taken from Table 3, and the data for In (3.34 K at 0.18 GPa) is taken from ref.^42^.

**Figure 5:**
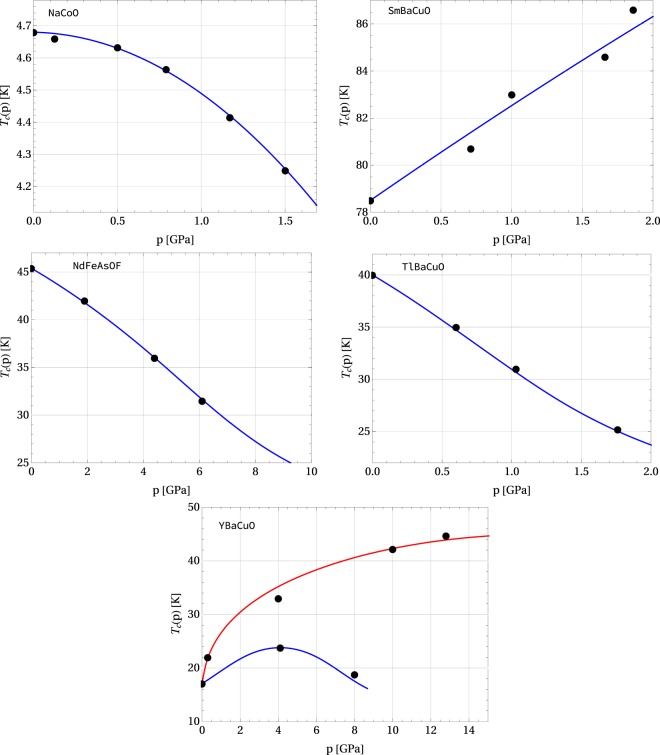
Critical temperature vs. pressure for selected superconductors: NaCoO, SmBaCuO, TlBaCuO, NdFeAsOF, YBaCuO. Black filled circles (●) indicate the experimental data according to refs^34–38^, respectively.

The experimental data and the corresponding values of the model parameters *x*_0_ and *χ* for the superconducting elements that we have discussed above are given in Tables 2 and 3. Figures 4 and 5 show the graphs of the critical temperature as a function of pressure for some selected superconductors. For some composed novel superconducting systems, such as *e.g*. SmBaCuO, NaCoO, TlBaCuO, NdFeAsOF, being of the type-(C) or (D), additional experimental data for ${ {\mathcal R} }_{1}$, ${ {\mathcal R} }_{2}$, and *c*_*p*_ at *p* = 0 is not fully available, but we are still able to estimate the parameters *x*_0_ and *χ* are from Eq. (9) for a few experimental points (*T*_*c*_(*p*), *p*), and then ${ {\mathcal R} }_{1}$ and ${ {\mathcal R} }_{2}$ can be determined from Eqs (3) and (5).

**Table 3:** Experimental data *T*_c_, ${ {\mathcal R} }_{1}$, ${ {\mathcal R} }_{2}$, and *c*_*p*_ for some superconducting materials. The value of model parameters |*x*_0_|, *χ* ${ {\mathcal R} }_{A}$, *χ*_*s*_, *κ*, ${T}_{{\rm{c}}}^{{\rm{\max }}\,/\,{\rm{\min }}}$, and *p*_*m*_ are found within the presented model. The type of the *T*_c_ vs pressure dependence is determined based on their values. ref.^19^ provides the following values for Thallium: ${T}_{{\rm{c}}}^{{\rm{\max }}}=2.395$ K and *p*_*m*_ = 0.195 GPa.

SC	*T*_c_	${{\boldsymbol{ {\mathcal R} }}}_{{\bf{1}}}$	${{\boldsymbol{ {\mathcal R} }}}_{{\bf{2}}}$	*c*_*p*_	|*x*_0_|	*χ*	*x*_0_	${{\boldsymbol{ {\mathcal R} }}}_{{\boldsymbol{A}}}$	*χ*_*s*_	*κ*	Type	${T}_{{\rm{c}}}^{{\rm{\max }}\,/\,{\rm{\min }}}$	*p*_*m*_
material	[K]	[1]	[1]	[K/GPa]		[1]	[1]	[1]	[1]	[GPa −^1^]		[K]	[GPa]
Hg (*α*)	4.156	4.6	2.4	−0.36	1.166	−2.787	−1.166	3.219	−3.025	0.196	(B)	1.83	59.6
Hg (*β*)	4.017	4.6	2.4	−0.44	1.166	−2.787	−1.166	3.219	−3.025	0.247	(B)	1.77	47.1
In	3.403	3.6	1.7	−0.436	0.546	0.521	0.546	3.808	−1.601	0.776	(C)		
Sn	3.732	3.5	1.6	−0.495	0.856	−1.334	−0.856	3.549	−2.135	0.253	(B)	2.20	33.9
Ta	4.301	3.6	1.6	−0.026	0.460	0.371	0.460	3.862	−1.504	0.0111	(C)		
Tl^(0)^	2.380	3.6	1.5	0.24	0.170	0.200	−0.170	3.891	−1.309	4.549	(A)	2.385	0.0377
Tl^(1)^	2.380	3.603	1.535	0.11	0.280	0.247	−0.280	3.948	−1.360	1.018	(A)	2.395	0.275

### Characteristic ratios and pressure coefficient

The hydrostatic pressure applied to a superconducting system does not only affect its critical temperature, but also other thermodynamic characteristics, such as the characteristic ratios. Consequently, Eq. (3) must be replaced by

$${ {\mathcal R} }_{1}(\chi ,{x}_{0},p)={ {\mathcal R} }_{1}^{\ast }\exp \{\chi [\frac{4}{\sqrt{{\mathrm{[4}\tau ({x}_{0}+\kappa p)]}^{2}+{ {\mathcal R} }_{1}^{2}(\chi ,{x}_{0},p)}}-\,\frac{\tanh \,[\tau ({x}_{0}+\kappa p)]}{\tau ({x}_{0}+\kappa p)}]\}.$$

Let us introduce yet another quantitative characteristics for a superconducting system subject to an external hydrostatic pressure: the pressure coefficient (at zero pressure) *c*_*p*_. This quantity is currently well-researched for many superconducting materials^1^. Within our model, as shown in ref.^33^, the pressure coefficient at zero pressure is given by

11 $${c}_{p}={\frac{d{T}_{{\rm{c}}}(\chi ,{x}_{0},p)}{dp}|}_{p=0}=\frac{\chi \kappa \,{T}_{{\rm{c}}}(\chi ,{x}_{0},\,\mathrm{0)}\,g({x}_{0})}{{x}_{0}\,[1+\chi \,g({x}_{0})]},$$

where $g(x)=(x\,{\cosh }^{-2}x-\,\tanh \,x)/x$ is an even function of *x*, and *g*(*x*) ≤ 0, *g*(0) = 0, *g*(*x*) → ∞ for |*x*| → ∞, and *g*(±1.639) = −0.4257 is its minimum value. Note that, ${c}_{p} < 0$ if ${x}_{0} < 0$ and $\chi  < 0$, *i.e*. for the type-(B) behavior, and ${c}_{p} > 0$ if ${x}_{0} > 0$ and $\chi  < 0$, *i.e*. for the type-(D) behavior, which is correct since the critical temperature is then a decreasing or an increasing function of pressure, respectively. However, for the type-(A) and the type-(C) behavior, the sign of *c*_*p*_ may change, although the critical temperature is an increasing or a decreasing function of pressure, respectively. Therefore, an additional condition $1+\chi \,g({x}_{0}) > 0$ must be imposed on the parameters *χ* and *x*_0_ resulting in the requirement that $\chi  < -\,1/g({x}_{0})$. Note that −1/*g*(*x*_0_) has a minimum of 2.349 for *x*_0_ = ±1.639, and it is proportional to |*x*_0_| if |*x*_0_| → ∞ and to |*x*_0_|^−2^ for |*x*_0_| → 0. Therefore, the condition $1+\chi \,g({x}_{0}) > 0$ does not impose any significant restrictions on the parameters *χ* and *x*_0_.

### Pressure-induced emergence of superconductivity

There are many materials that undergo a superconducting phase transition under high pressure^5,39,40^, with some examples listed in Tables 4 and 5. Please note that the experimental data originates from two different sources, and therefore, although both may list the same elements, there is some variation in the experimentally measured values.

**Table 4:** Elements undergoing a transition to the superconducting state under high pressure^5^.

Element	Li	B	O	Si	P	S	Ca	Sc	Fe	Ge	As
*T*_c_ [K]	20	11.2	0.6	8.5	18	17	15	0.34	2	5.4	2.7
*p* [GPa]	50	250	120	12	30	160	150	21	21	11.5	24
Element	Se	Br	Sr	Y	Sb	Te	I	Cs	Ba	Bi	Ce
*T*_c_ [K]	7	1.4	4	2.8	3.6	7.4	1.2	1.66	5	8.7	1.75
*p* [GPa]	13	150	50	15	8.5	35	25	8	20	9	5

**Table 5:** Elements undergoing a transition to the superconducting state under high pressure^39,40^. The critical temperature for Li is estimated at 0.0004 K, so it should be left out of analysis here.

Element	Li	B	O	Si	P	S	Ca	Sc	Fe	Ge	As	Se
*T*_c_ [K]	14	11	0.6	8.2	13	17.3	29	19.64	2.1	5.35	2.4	8
*p* [GPa]	30	250	100	15.2	30	190	217	106	21	11.5	32	150
Element	Br	Sr	Y	Sb	Te	I	Cs	Ba	Bi	Ce	Eu	Lu
*T*_c_ [K]	1.4	7	19.5	3.9	7.5	1.2	1.3	5	8.5	1.7	2.75	12.4
*p* [GPa]	100	50	115	25	35	25	12	18	9.1	5	142	174

We will now demonstrate how the developed model, with a single fluctuation of height of *χ* in the density of state, shifted a distance *x*_0_ from the Fermi level, can be used to to explain the effect of emergence of superconductivity in a system under external pressure. First of all, one should remember that the superconducting state is realized in the system if Eq. (3) has stable solutions ${ {\mathcal R} }_{1}(\chi ,{x}_{0})$. However for a fixed *x*_0_, when $\chi  < {\chi }_{s}$ in general, or rather $\chi  < {\chi }_{m}$, superconductivity is suppressed in the system, as discussed in Section 3.3.

However, after applying an external pressure, the parameter *x*_0_ is replaced by ${\tilde{x}}_{0}=\tau ({x}_{0}+\kappa p)$, as discussed at the beginning of the present section. Hence, when it reaches the value ${\tilde{x}}_{0} > |{x}_{0}|$ such that ${\chi }_{s}({\tilde{x}}_{0}) > {\chi }_{s}({x}_{0})$, superconductivity should appear.

Let us consider a few more specific examples, with some sets of parameters *x*_0_ and *χ* characterizing a single fluctuation in the density of states.

A Let *x*_0_ = 0 and *χ* = −0.6. Superconductivity is not realized in this system, because *χ*_*m*_(0) = −0.579 and $-0.6 < {\chi }_{m}\mathrm{(0)}$. However, after applying an external pressure, *x*_0_ is replaced by ${\tilde{x}}_{0}=\tau \kappa p$, and when ${\tilde{x}}_{0}$ exceeds 0.1082 the superconducting state can be realized in the system for appropriate temperatures. This is because −0.6 ≥ *χ*_*m*_(0.1082) and ${ {\mathcal R} }_{1}(-\,\mathrm{0.6,0.1082)}=2.275$, and while ${\tilde{x}}_{0}=\tau \kappa p=0.4$, then ${ {\mathcal R} }_{1}(-\mathrm{0.6,0.4)}=3.1768$.
B Let *x*_0_ = 0.2 and *χ* = −0.67. Superconductivity is suppressed in this system as well, because *χ*_*m*_(0.2) = −0.654. By placing the system under an external pressure, ${\tilde{x}}_{0}=\tau ({x}_{0}+\kappa p)=0.4$ and superconductivity can be realized in the system at appropriate temperatures, because ${ {\mathcal R} }_{1}(-\mathrm{0.67,0.4)}=3.080$, while ${\tilde{x}}_{0}=\tau ({x}_{0}+\kappa p)=0.7$ then ${ {\mathcal R} }_{1}(-\mathrm{0.67,0.7)}=3.437$.
C If *x*_0_ = −0.2 and *χ* = −0.67, superconductivity appears in this system as in previous example, *i.e*. for ${\tilde{x}}_{0}=\tau ({x}_{0}+\kappa p)=0.4$ or ${\tilde{x}}_{0}=\tau ({x}_{0}+\kappa p)=0.7$. However, depending on the values of *κ* and *τ*, the threshold values of the pressure can differ significantly.
D If the pressure increases, and ${\tilde{x}}_{0}=\tau ({x}_{0}+\kappa p)$ reaches values close to ${x}_{0}^{\ast }=\pm 0.879077\ldots$, solutions to Eq. (3) may fall into the instability region, and superconductivity is suppressed.

**Figure 6:**
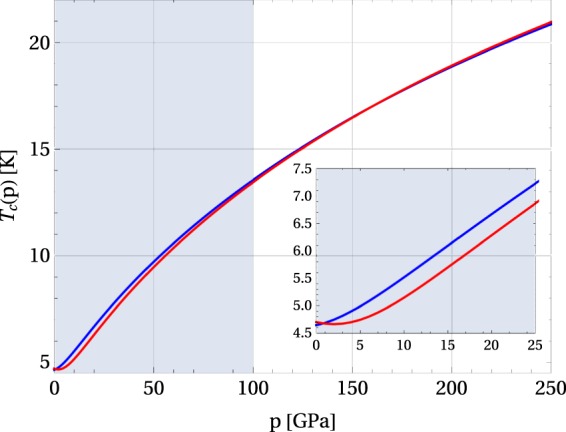
Two possible forms of superconductivity appearing in sulphur under pressure, corresponding to type-(B) (red curve) or type-(D) behavior (blue curve). The shaded area marks the unstable region, corresponding to the range of pressure where superconductivity is suppressed. The system becomes superconducting as soon as the pressure exceeds the threshold value beyond the shaded area^6^.

The phenomenon of pressure-induced superconductivity can be further illustrated with the example of the sulphur, using results presented in refs^5,6^. The sulphur belongs to a class of materials, where superconductivity appears only under pressure. Finding the values of model parameters corresponding to the experimental data, we have *x*_0_ = ±0.1 and *χ* = −2.335. Consequently, since *χ*_*m*_(±0.1) = −0.597 and $\chi  < {\chi }_{m}(\pm \mathrm{0.1)}$, superconductivity does not appear. However, if the external pressure is applied and reaches the value of 100 GPa, the system becomes superconducting, which indicates that ${\chi }_{m}({\tilde{x}}_{0})=-\,2.335$. After estimating *T*_c_(*p*) for *p* = 0, 100, 160 and 250 GPa by fitting the numerical results presented in Fig. 6, one can find the value of *κ* from the relations

$${\chi }_{m}({\tilde{x}}_{0})=-\,2.335\,{\rm{and}}\,{\tilde{x}}_{0}=\frac{{T}_{{\rm{c}}}\mathrm{(0)}}{{T}_{{\rm{c}}}\mathrm{(100)}}(\pm 0.1+\kappa 100).$$

Using the values of the critical temperature given in Table 6, and calculating ${\tilde{x}}_{0}=0.940$ from Eq. (9), one finds *κ* = 0.0279 GPa^−1^ and *κ* = 0.0264 GPa^−1^ for the case *x*_0_ = −0.1 and *x*_0_ = 0.1, respectively.

**Table 6:** Parameters ${\tilde{x}}_{0}=\tau ({x}_{0}+\kappa p)$ and *χ*_*m*_ for sulphur under high pressure estimated based on refs^5,6^. The two cases refer to (I) *x*_0_ = −0.1, *χ* = − 2.335 and *κ* = 0.0279 GPa^−1^; (II) *x*_0_ = 0.1, *χ* = −2.335 and *κ* = 0.0264 GPa^−1^.

p	Case I			Case II		
	T_c_(p)	${\tilde{{\boldsymbol{x}}}}_{{\bf{0}}}$	χ_m_	T_c_(p)	${\tilde{{\boldsymbol{x}}}}_{{\bf{0}}}$	χ_m_
[GPa]	[K]	[1]	[1]	[K]	[1]	[1]
0	4.70	−0.1	−1.289	4.65	0.1	−1.289
100	13.4	0.940	−2.335	13.5	0.940	−2.335
160	17.0	1.206	−3.173	17.0	1.128	−2.893
250	21.0	1.542	−4.778	20.9	1.441	−4.221

The discussion shows that for systems where superconductivity is purely pressure-induced, the superconducting phase is not realized if the pressure is below a certain threshold value, since it is unstable there. In the particular case of the sulphur, assuming it to be a type-(B) or a type-(D) material leads to similar conclusions. And in both cases the critical temperature always increases with the pressure. Therefore, we should also expect that there are some materials with pressure-induced superconductivity, for which the critical temperature is a decreasing function of the pressure.

As a final remark to the present section, let us emphasize that one should keep in mind that a high pressure can change the structure of the material. These structural changes may imply a new form of the dispersion relation and hence a different form of the fluctuation considered in this simple model. Consequently, the material can exhibit different properties.

## Model Parameters and Experimental Data

### Determination of model parameters from measurement data

Experimental data, such as the critical temperature *T*_c_, the characteristic ratios ${ {\mathcal R} }_{1}$ and ${ {\mathcal R} }_{2}$, as well as the pressure coefficient at zero pressure *c*_*p*_, are widely available in the literature for various superconducting systems^5,14–21^. However, as we have already mentioned before, the experimentally found values may vary by several percent as a result of differences in characterization techniques and methods.

Nevertheless, we still can use the experimental data to find the values of our model parameters: |*x*_0_| from Eq. (5), *χ* from Eq. (3), ${ {\mathcal R} }_{A}$ from Eq. (7), *χ*_*s*_ from Eq. (8) *κ* from Eq. (11). Note that a system with ${c}_{p} < 0$ and $\chi  < 0$ must be of the type (B), and for ${c}_{p} > 0$ and $\chi  > 0$ it must be of the type (A), hence ${x}_{0} < 0$, and additional measurable parameters, *i.e*. the minimal or maximal critical temperatures ${T}_{{\rm{c}}}^{{\rm{\max }}\,/\,{\rm{\min }}}$ at the pressure *p*_*m*_ = −*x*_0_/*κ* are given by

12 $${T}_{{\rm{c}}}^{{\rm{\max }}\,/\,{\rm{\min }}}={T}_{{\rm{c}}}\exp [\chi (1-\frac{\tanh \,{x}_{0}}{{x}_{0}})]\,.$$

The values of these parameters calculated for some superconducting elements are given in Table 3, with the data for *T*_c_ and *c*_*p*_ taken from ref.^19^.

### Compatibility criterion for experimental data

The developed model allows us to establish a very useful criterion for testing compatibility of experimental data. The criterion verifies whether the estimated values of ${ {\mathcal R} }_{1}$ and ${ {\mathcal R} }_{2}$ are compatible with the data for *T*_c_ and ${T}_{{\rm{c}}}^{{\rm{\max }}\,/\,{\rm{\min }}}$ for type-(A) or type-(B) superconductors.

The criterion is established as follows: For a superconducting system, the values of ${ {\mathcal R} }_{1}$ and ${ {\mathcal R} }_{2}$ found experimentally allow us to derive the parameter *x*_0_ from Eq. (5), and then by substituting ${ {\mathcal R} }_{1}$ and *x*_0_ into Eq. (3), we can find the corresponding value of the parameter *χ*. On the other hand, for the same superconductor (either type-(A) or type-(B)) after substituting their critical temperatures *T*_c_ and ${T}_{{\rm{c}}}^{{\rm{\max }}\,/\,{\rm{\min }}}$ into Eq. (12), the parameter *χ* can calculated again using an alternative method. These both values of the parameter *χ* estimated from the experimental data should be identical. Therefore, by comparing them we can directly evaluate the precision of experimental measurements.

Referring to the experimental data for superconducting materials discussed in this paper as examples, it is worth to notice that the values of *χ* obtained for the tantalum are equal to 0.345, as given in Table 2, and 0.344 when it is found from Eq. (12). The two values of *χ* obtained for the thallium are equal to 0.200 or 0.247 for Tl^(0)^ and Tl^(1)^ (Table 3), and 0.660 or 0.248 when it is found from Eq. (12). Finally, the values of the same parameter found for the niobium are equal to 0.689 (Table 2) for Nb^(1)^, and 0.689 as well, if found from Eq. (12).

A brief comparison of the values of *χ* for the thallium, given in Table 3, immediately indicates that the estimates of parameters presented in ref.^19^ as Tl^(0)^ are not correct, whereas those given as Tl^(1)^ pass the compatibility test. Moreover, the experimental data for the niobium presented in Table 2, *i.e*. Nb^(0)^, Nb^(1)^, Nb^(2)^, are not compatible, and one should definitely reject the claim that it is a type-(D) material.

With the example of the thallium it has been shown that a simple estimate of the experimental data given in ref.^19^, that is the line Tl^(0)^ is in contradiction with other parameters of the model. However, if the experimental data were slightly different, but still within the accuracy of the measurements, and the values of *c*_*p*_ and *p*_*m*_ were estimated again, the received results shown as the line Tl^(1)^ would coincide quite well with the points showing the experimental data. Therefore, the postulated values of *c*_*p*_ = 0.24 K/GPa and *p*_*m*_ = 0.195 GPa^19^ should rather be replaced by *c*_*p*_ = 0.11 K/GPa and *p*_*m*_ = 0.275 GPa.

A careful analysis of the examples of Tl^(0)^ and Tl^(1)^ points to the fact that the simple model is sensitive to minor changes in the output parameters. At the same time, it imposes some conditions on the postulated parameters, such as on the values of *c*_*p*_ and *p*_*m*_, so that the results can match the experimental data in the best possible way.

## Conclusions

In our previous work^33^, we have identified and discussed four universal types of the response of superconducting systems to an external high pressure, in terms of the dependence of the critical temperature on pressure. In the present paper, within that model^33^, we have discussed further pressure-related properties of some specific superconducting materials and referred them to available experimental data. The wide range of numerical results indicates that experimental data can be successfully used to find the critical temperature as a function of pressure, and discuss other properties of superconducting systems under high pressure. It should be emphasized that the presented approach contains a significantly simplified density of states, therefore it can be applied to complex superconductors only to a limited extent. Nevertheless, the available experimental data support the observation that the dependence of the critical temperature on external pressure can be identified as being one of four general types: (A), (B), (C), or (D).

Worth noting is that the results obtained within our model are sensitive to the uncertainty of experimental data, which is often not analyzed in papers presenting experimental results. Therefore, even for the same superconducting system and the same parameter, experimental results obtained using different measurement techniques, provide values that may sometimes differ to a relatively large extent. This dispersion in the experimental data can lead to contradictory conclusions, as *e.g*. for the niobium.

For the same reason, we did not identify the type of some superconductors given in Table 2. For instance, in the case of the aluminium, using the compatibility criterion formulated in the paper, we have found the data given in the literature^20,39,40^ to be incompatible, although according to refs^5,41^ the aluminium should be of a type-(C) system. We also doubt that the transition temperature estimated for Lithium is *T*_c_ = 0.0004 K^39,40^, since the critical temperature should be enhanced by ca. 3.5 × 10^4^ times. Hence, the proposed scenario of superconductivity appearing under pressure seems to be more convincing.

As a final remark, let us emphasize that the presented simplified model is based on the assumption that the crystal structure of a superconductor is stable under high pressure. In general, however, high pressure can induce structural phase transitions in some materials, and properties of such systems may change drastically. Consequently, the one-particle dispersion relation *ξ*_***k***_ assumes a new, stable form, and if the superconductivity is not suppressed, the new system can be analyzed again within the conformal transformation method. However, compared to the original system, before the structural phase transition has taken place, the system with a new structure may reveal quite different properties. In particular, one type of the dependence of the critical temperature on pressure might be replaced by another, when the pressure is being increased. Therefore, one can expect that in quasi-two-dimensional systems, such as films of elements discussed in ref.^5^, superconductivity appears due to an essential change in the one-particle dispersion relation *ξ*_***k***_. Such systems can be considered within the developed model as well^13,24,33^.

## Data Availability

All data generated or analyzed during this study are included in this published article.
